# Seizure Mimics as the Presenting Feature of a Functional Metastatic Pancreatic Neuroendocrine Tumour in a Patient With Pre-existing Epilepsy: A Diagnostic Challenge

**DOI:** 10.7759/cureus.110100

**Published:** 2026-06-02

**Authors:** Itizaz Hayat, Taimoor Tahir, Maheen Omer, Ibrahim Elzeiny, May Loo

**Affiliations:** 1 Internal Medicine, Queen Elizabeth Hospital, Gateshead, GBR; 2 Medicine, Kuwait Teaching Hospital, Peshawar, PAK; 3 Medicine, Darlington Memorial Hospital, Darlington, GBR; 4 Endocrinology and Diabetes, Queen Elizabeth Hospital, Gateshead, GBR

**Keywords:** diazoxide, endogenous hyperinsulinaemia, hypoglycaemia, insulinoma, lanreotide, neuroglycopenia, pancreatic neuroendocrine tumours, seizure mimic

## Abstract

Insulinomas and functional pancreatic neuroendocrine tumours (pNETs) are rare but important causes of endogenous hyperinsulinaemic hypoglycaemia whose neuroglycopenic manifestations can closely mimic neurological disorders, leading to considerable diagnostic delay. We present the case of a 74-year-old man with epilepsy, seizure-free for 11 years on carbamazepine, and a background of metastatic well-differentiated grade 3 pNET (Ki-67: 40-50%), who was admitted with a 2-3-week history of nocturnal confusion, cold sweats, nightmares, increasing agitation, and an episode of abnormal limb movements. The presentation was initially attributed to possible seizure recurrence or delirium. Routine investigations, including CT brain, carbamazepine levels, and type 1 antineuronal nuclear autoantibody antibody testing, were unremarkable, and blood glucose on admission was normal. The correct diagnosis was reached only during a witnessed ward episode on day two, when capillary blood glucose was found to be critically low and symptoms resolved immediately with intravenous dextrose. Subsequent biochemical evaluation demonstrated markedly elevated serum insulin (42 µU/L; reference: 2-24 µU/L), C-peptide (2,464 pmol/L; reference: <90 pmol/L), and on formal supervised 72-hour fast, which provoked symptomatic hypoglycaemia after just three hours, insulin was 180 µU/L, proinsulin was 730 pmol/L (reference: <10 pmol/L), and C-peptide was 2,136 pmol/L, with a negative sulphonylurea screen, fulfilling the diagnostic criteria for endogenous hyperinsulinaemia. Somatostatin receptor scintigraphy (single-photon emission computed tomography-computed tomography) identified multifocal hepatic metastases and focal uptake in the pancreatic tail, confirming an insulin-secreting primary. The patient was managed with diazoxide and monthly lanreotide but experienced rapid deterioration and died on palliative care. This case underscores the need to consider a functional pNET as a cause of seizure-like episodes even in patients with established epilepsy, and highlights that capillary blood glucose measurement during any witnessed neurological episode is a simple but potentially life-saving clinical step that should never be omitted.

## Introduction

Pancreatic neuroendocrine tumours (pNETs) constitute a heterogeneous group of neoplasms arising from the endocrine cells of the pancreas. When functional, they secrete hormones in excess, producing characteristic clinical syndromes. Insulinomas, the archetypal functioning pNET, are the most common hormone-secreting tumours of the gastrointestinal tract, with an annual incidence of approximately 1-3 cases per million [1]. The vast majority are benign, solitary, and less than 2 cm in diameter; malignant insulinomas with distant metastases at presentation are reported in approximately 5-14% of cases [2,3].

The clinical presentation of insulinoma is governed by the consequences of inappropriate insulin secretion during fasting or exertion. Symptoms broadly fall into two categories: adrenergic features (palpitations, diaphoresis, tremor) and neuroglycopenic features (confusion, bizarre behaviour, amnesia, and seizure-like activity) [4]. The insidious, episodic, and non-specific nature of neuroglycopenia renders it one of medicine’s most frequently missed diagnoses. It has been estimated that it takes an average of 1.5 years from symptom onset to correct diagnosis, and more than half of patients with neuroglycopenic presentations are initially diagnosed with a primary neuropsychiatric disorder, most commonly epilepsy [1,4,5].

The diagnostic challenge is compounded further when a patient carries an established neurological diagnosis. In such cases, new symptoms are liable to be attributed to the existing condition, narrowing the differential and delaying the recognition of an underlying metabolic aetiology. We present the case of a 74-year-old man with long-standing, well-controlled epilepsy, in whom nocturnal episodes of confusion, diaphoresis, and abnormal limb movements were initially attributed to seizure recurrence. The true aetiology was eventually identified as endogenous hyperinsulinaemic hypoglycaemia arising from a functional metastatic pNET, a tumour whose existence was already known, yet whose secretory activity had not previously been recognised.

This case illustrates a convergence of several factors that together created a perfect diagnostic storm: an atypical presentation in an elderly patient, a competing established diagnosis, a known underlying malignancy not previously considered functional, and symptoms occurring in a context (overnight and early morning) that might naturally prompt consideration of seizure activity. We aim to highlight the critical importance of glucose measurement during any witnessed neurological episode and to discuss the biochemical and imaging pathways to the diagnosis of a functional pNET.

## Case presentation

A 74-year-old man was admitted to the geriatrics ward with a 2-3-week history of nocturnal confusion, cold sweats, and vivid nightmares. These episodes had worsened progressively, evolving to include increased aggression, severe disorientation, and, on one occasion, abnormal limb movements witnessed by his family. All episodes occurred predominantly overnight or in the early morning. During the day, the patient appeared entirely well and denied any ongoing symptoms.

His past medical history was significant for two conditions directly relevant to this presentation. First, he had a longstanding diagnosis of epilepsy, for which he had been taking carbamazepine for many years. Crucially, he had been entirely seizure-free for 11 years on this treatment. Second, he had been under active oncological follow-up for a metastatic, well-differentiated, grade 3 pNET (Ki-67 index: 40-50%), first identified in September 2023.

The oncological history is worth recounting in detail. His initial presentation in September 2023 was with several weeks of nausea, vomiting, and weight loss without diarrhoea. Upper and lower gastrointestinal endoscopy at that time was unremarkable. A contrast-enhanced CT of the thorax, abdomen, and pelvis demonstrated multiple hepatic lesions with morphology and enhancement patterns highly suggestive of metastatic neuroendocrine tumour; no primary pancreatic lesion was identified on that initial scan (Figure 1).

**Figure 1:**
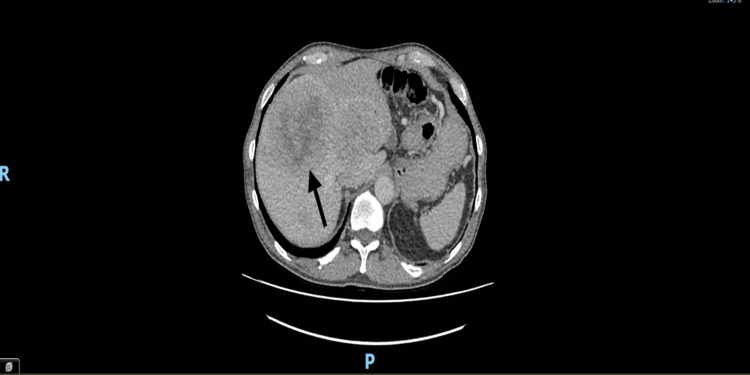
Contrast-enhanced CT of the abdomen demonstrating multiple hepatic lesions (arrow) with enhancement patterns consistent with metastatic neuroendocrine tumour. No primary pancreatic lesion was identified on this initial imaging.

An ultrasound-guided core biopsy of a liver lesion in November 2023 confirmed metastatic well-differentiated neuroendocrine tumour, WHO grade 3. Immunohistochemistry demonstrated PAX8 positivity, consistent with a pancreatic primary, along with positive staining for chromogranin and synaptophysin. The Ki-67 index was 40-50%. Importantly, the tumour had not been considered functional at the time of diagnosis; there was no biochemical evaluation for hormonal hypersecretion, and no features of hypoglycaemia were present.

He was commenced on systemic chemotherapy with capecitabine and temozolomide, which produced a partial radiological response by September 2024, with resolution of his initial gastrointestinal symptoms. However, follow-up imaging in December 2024 showed significant progression of hepatic metastases, and chemotherapy was reinitiated in January 2025.

On the current admission, clinical examination was unremarkable. There was no focal neurological deficit. Routine blood tests showed mildly deranged liver function (gamma-glutamyl transferase: 250 U/L) in keeping with his known hepatic disease, and a carbamazepine level of 4.2 mg/L, within the accepted therapeutic range. Blood glucose checked on admission was normal. A contrast-enhanced CT of the brain showed no acute intracranial pathology. Type 1 antineuronal nuclear autoantibody testing was negative, making autoimmune encephalitis less likely.

The patient remained convinced he was experiencing seizure recurrence, and the initial clinical impression was one of possible delirium or atypical seizures in the context of his oncological illness. The apparently normal blood glucose on admission, coupled with the pre-existing epilepsy diagnosis, reasonably directed attention away from a metabolic aetiology.

On day two of admission, nursing staff witnessed an episode of abnormal behaviour with associated limb movements. An ABCDE assessment was unremarkable, except for critically low capillary blood glucose. The episode resolved rapidly and completely with intravenous 10% dextrose. Blood samples for serum insulin and C-peptide were taken at the time of the hypoglycaemic episode, before glucose correction. The patient was commenced on low-dose oral prednisolone as a temporary measure to maintain euglycaemia.

The insulin level returned at 42 µU/L (reference range: 2-24 µU/L) and the C-peptide at 2,464 pmol/L (reference range: <90 pmol/L). The significant elevation of both insulin and C-peptide in the context of documented symptomatic hypoglycaemia was consistent with endogenous hyperinsulinaemia, effectively excluding exogenous insulin administration as a cause.

A formal supervised 72-hour fast was performed to validate these findings in a controlled setting. The patient developed symptomatic hypoglycaemia with a venous plasma glucose of 1.8 mmol/L after just three hours of fasting. Blood samples obtained at the time of hypoglycaemia confirmed insulin of 180 µU/L (reference: 2-24 µU/L), proinsulin of 730 pmol/L (reference: <10 pmol/L), C-peptide of 2,136 pmol/L (reference: <90 pmol/L), and a negative sulphonylurea screen. The markedly elevated proinsulin is notable and has been associated with malignant insulinoma behaviour [2]. Beta-hydroxybutyrate was appropriately suppressed, further confirming insulin-driven hypoglycaemia. Whipple’s triad was fully satisfied: symptomatic hypoglycaemia, documented low blood glucose, and rapid resolution of symptoms with glucose administration [4].

The patient was discharged on diazoxide while awaiting specialist endocrinology review. An octreotide challenge test was subsequently performed in the outpatient endocrinology setting, and a diagnosis of insulin-secreting pancreatic NET was formally confirmed in February 2025.

Functional imaging with somatostatin receptor scintigraphy (single-photon emission computed tomography-computed tomography (SPECT-CT)) performed on 11 March 2025 demonstrated multifocal large-volume increased tracer uptake throughout the liver, consistent with widespread hepatic metastases, and focal marked uptake in the tail of the pancreas, consistent with the primary tumour. This established the diagnosis of a functional, insulin-secreting, metastatic pNET with the primary in the pancreatic tail, a lesion not visualised on the original CT performed at the time of diagnosis.

The patient was managed with escalating doses of diazoxide and monthly subcutaneous lanreotide injections. Unfortunately, he was readmitted shortly after with a mechanical fall, a concurrent lower respiratory tract infection, recurrent hypoglycaemia, and severe hyponatraemia. Despite critical care support, his clinical condition did not improve, and after careful discussion with the patient and his family, he was transitioned to palliative care. He passed away during that admission.

## Discussion

This case illustrates a rare but instructive diagnostic scenario in which a functional pNET producing endogenous hyperinsulinaemia presented as seizure-like episodes in a patient with pre-existing, well-controlled epilepsy. Several lessons emerge that are broadly applicable to both general medicine and neurology practice.

The phenomenon of insulinoma masquerading as epilepsy is well documented, albeit infrequently reported. Graves et al. described a landmark case of a 44-year-old woman with multiple insulinomas who had been managed with escalating doses of multiple antiepileptic drugs over 4.5 years before the correct diagnosis was reached. The diagnosis was established only when glucose was checked during a witnessed attack on an inpatient electroencephalogram monitoring unit and found to be 0.8 mmol/L, a stark reminder of the diagnostic value of a simple point-of-care glucose measurement [5]. In a retrospective study of 59 patients with histologically confirmed islet cell adenomas reviewed in that same report, 39% had originally been diagnosed with a seizure disorder [5]. Fayyaz et al. similarly described a woman in her 60s who was commenced on levetiracetam for complex partial seizures before hypoglycaemia was identified on a subsequent presentation, ultimately leading to the diagnosis of a 16 mm insulinoma in the uncinate process of the pancreas [1].

What distinguishes the present case from those previously described is the presence of a known underlying pNET that had not been considered functional. This adds a further layer of diagnostic complexity: not only did the pre-existing epilepsy diagnosis provide a competing explanation for the neurological symptoms, but the known malignancy had previously been evaluated and treated without any recognition of insulin hypersecretion. This is biologically plausible, as pNETs can acquire or develop new secretory capabilities over time, or a functionally active subclone may become dominant during tumour progression. The significant increase in tumour burden evident on December 2024 imaging preceded the onset of hypoglycaemic symptoms, suggesting that progressive hepatic involvement may have driven the emergence of clinically relevant insulin hypersecretion.

The nocturnal and early-morning predilection of the patient’s symptoms is a clinically important feature that, in retrospect, is highly characteristic of fasting-state hypoglycaemia. The brain is exquisitely sensitive to glucose deprivation, and the cortical and subcortical dysfunction produced by neuroglycopenia can convincingly simulate a range of neurological and psychiatric conditions, including delirium, transient ischaemic attacks, complex partial seizures, and dementia [4,5]. The key differentiating feature, that symptoms reliably occur in the fasting state and resolve with carbohydrate ingestion, may be overlooked if not specifically sought in the history, particularly in elderly patients who may not recognise or articulate the relationship between food intake and symptom relief.

The biochemical profile obtained during the spontaneous hypoglycaemic episode and subsequently during the supervised fast was diagnostically unambiguous. Endogenous hyperinsulinaemia is defined by inappropriately elevated insulin (typically >3 mIU/L or >18 pmol/L) and C-peptide (>0.2 nmol/L) in the context of fasting plasma glucose below 2.5-3.05 mmol/L [6]. In this patient, insulin and C-peptide levels were elevated many-fold above their reference ranges at the time of symptomatic hypoglycaemia, consistent with the findings described by Sandoval et al. in a malignant insulinoma, where insulin was elevated 32-fold above diagnostic thresholds [2]. The disproportionate elevation of proinsulin (730 pmol/L against a reference of <10 pmol/L) is particularly noteworthy; elevated proinsulin has been associated with malignant insulinoma and may reflect aberrant post-translational processing within dedifferentiated or high-grade tumour cells [2].

The negative sulphonylurea screen was an essential component of the diagnostic evaluation, given that sulphonylurea ingestion produces an identical biochemical pattern of elevated insulin and C-peptide with suppressed glucose. Exogenous insulin administration was effectively excluded by the elevated C-peptide, which would be suppressed in the setting of exogenous insulin use.

Somatostatin receptor scintigraphy (SPECT-CT) played a pivotal role in confirming the functional nature of the tumour and identifying the primary pancreatic lesion, which had not been visible on conventional CT for over a year. This underscores the complementary role of functional imaging in the evaluation of pNETs. More than 60-100% of pNETs express somatostatin receptors, enabling targeted radionuclide imaging with high sensitivity, particularly for small or metabolically active tumours [4]. The identification of the pancreatic tail lesion in this case demonstrates that somatostatin receptor-based imaging should be considered early in the workup of suspected functional pNET, particularly when anatomical imaging is non-contributory.

Management of metastatic insulin-secreting pNET is challenging and is typically multimodal. The primary therapeutic objective in the short term is the prevention of hypoglycaemia. Frequent small meals and complex carbohydrates represent the simplest first-line dietary intervention [1]. Diazoxide, a benzothiadiazine derivative that inhibits insulin secretion by opening ATP-sensitive potassium channels in pancreatic beta cells, is the first-line pharmacological agent for glycaemic control [2,7]. Its principal adverse effects include fluid retention and hirsutism; in the present case, the patient required ongoing dose titration. Somatostatin analogues, including octreotide and lanreotide, reduce insulin secretion in tumours expressing somatostatin receptor subtypes 2A and 5, and may also exert an antiproliferative effect [2,4]. Glucocorticoids act by increasing insulin resistance and stimulating hepatic gluconeogenesis and were used as a bridging measure in this patient. The combination of diazoxide and lanreotide, as employed here, mirrors the pharmacological approach described by Sandoval et al. in their management of a malignant insulinoma with functioning liver metastases [2].

Debulking surgery, transarterial chemoembolisation, and radiofrequency ablation of liver metastases may also be considered in selected patients to reduce tumour bulk and alleviate hormonal hypersecretion [2]. In this patient, such interventions were not feasible given the extent of hepatic disease and his deteriorating performance status. The rapid clinical decline following the correct diagnosis reflects the aggressive biology of a WHO grade 3 pNET with extensive metastatic disease, and serves as a reminder that the establishment of a correct diagnosis does not necessarily translate into a favourable prognosis.

The management of multiple endocrine neoplasia type 1 (MEN1)-related pNETs offers an instructive comparator with respect to prognosis. Van Beek et al. demonstrated in a large multicentre cohort that MEN1-related insulinomas had a significantly more favourable liver metastasis-free survival compared to non-functioning pNETs, with an estimated 10-year survival of 87% versus 63%, respectively, a distinction driven in part by earlier presentation due to symptomatic hypoglycaemia [8]. The present case represents the opposite end of the spectrum: a sporadic, functionally silent pNET that became hormonally active at a late stage of disease, by which point the burden of metastatic disease precluded curative intervention.

From a nosological perspective, this case also highlights that a pNET may remain non-functional for a prolonged period before acquiring or manifesting hormonal activity, and that the onset of hypoglycaemia in a patient with a known pNET should always prompt urgent biochemical evaluation for insulin hypersecretion, regardless of the clinical context.

## Conclusions

We present a diagnostically challenging case in which a metastatic functional pNET producing endogenous hyperinsulinaemia presented with nocturnal neuroglycopenic episodes misattributed to seizure recurrence in an elderly man with established epilepsy. The correct diagnosis was reached only when blood glucose was measured during a witnessed episode, a step that should be routine in any patient with unexplained transient neurological dysfunction, regardless of prior diagnoses. Key clinical lessons from this case include (1) neuroglycopenic symptoms may convincingly mimic epileptic seizures and other neurological disorders; (2) a pre-existing neurological diagnosis should not preclude consideration of a metabolic aetiology; (3) a known pNET that was not initially considered functional may become hormonally active during disease progression; (4) the fasting-state predilection of symptoms, particularly overnight and in the early morning, is a clinically important clue to hypoglycaemic aetiology; and (5) early glucose measurement during witnessed episodes and prompt biochemical evaluation of hyperinsulinaemia are essential to avoid prolonged diagnostic delay and the potential for irreversible neurological harm from uncontrolled hypoglycaemia.
